# Understanding HIV-Exposed Uninfected Children: A Narrative Review

**DOI:** 10.3390/v17030442

**Published:** 2025-03-19

**Authors:** Martina Salvi, Benedetta Fioretti, Maria Alberti, Irene Scarvaglieri, Stefania Arsuffi, Giorgio Tiecco, Francesco Castelli, Eugenia Quiros-Roldan

**Affiliations:** Department of Clinical and Experimental Sciences, SD of Infectious and Tropical Diseases, University of Brescia and ASST Spedali Civili di Brescia, 25123 Brescia, Italy; m.salvi026@unibs.it (M.S.); b.fioretti@unibs.it (B.F.); m.alberti035@unibs.it (M.A.); i.scarvaglieri@studenti.unibs.it (I.S.); s.arsuffi@unibs.it (S.A.); g.tiecco@unibs.it (G.T.); francesco.castelli@unibs.it (F.C.)

**Keywords:** HIV-exposed uninfected children, HEU, HIV maternal exposure, cardiovascular, bone, growth, neurocognitive, inflammation

## Abstract

The widespread implementation of antiretroviral therapy has significantly reduced HIV-related mortality and mother-to-child transmission. Despite being HIV-uninfected, HIV-exposed children (HEU) seem to face heightened risks of immune dysfunction, cardiometabolic diseases, growth delays, reduction in bone mineral density, and neurocognitive impairments compared to HIV-unexposed uninfected peers. These vulnerabilities can be attributed to maternal immune dysregulation during pregnancy, antiretroviral (ART) toxicity, HIV exposure, and adverse socioeconomic and nutritional environments. Emerging evidence highlights the impact of antiviral therapy exposure, particularly tenofovir disoproxil fumarate, on HEU mitochondrial dysfunction, bone resorption, neurocognitive delays, and zidovudine on cardiac abnormalities. This narrative review explores the multisystem effects of ART exposure in HEU children, focusing on immune function, neurodevelopment, cardiovascular health, growth, and bone metabolism. By synthesizing findings from diverse studies, the review aims to provide a comprehensive understanding of the potential risks associated with ART regimens and identify future research priorities to improve outcomes for HEU children.

## 1. Introduction

The global scale-up of antiretroviral therapy (ART) has been a cornerstone in reducing HIV-related mortality and preventing mother-to-child transmission of HIV. This success has resulted in a growing population of HIV-exposed but uninfected (HEU) children who, despite not contracting the virus, seem to face unique health challenges. As of 2023, an estimated 15 million HEU children live worldwide, predominantly in sub-Saharan Africa, where the burden of HIV remains highest [1]. These children are born into environments characterized by socioeconomic adversity, maternal immune activation, and in utero exposure to ART, all of which contribute to their complex health profiles.

Emerging evidence suggests that HEU children are at a greater risk of immune dysfunction, severe infections, growth delays, neurocognitive impairments, and metabolic abnormalities compared to HIV-unexposed uninfected children (HUU) [2]. These vulnerabilities are believed to stem from a combination of factors, including maternal immune dysregulation during pregnancy, ART toxicity, and environmental determinants such as nutrition and socioeconomic status [3].

A central focus of current research is the long-term impact of ART exposure, particularly tenofovir disoproxil fumarate (TDF), on bone health, immunity, and metabolism [4,5] and zidovudine (ZDV) on cardiac development in HEU children. Additionally, concerns about neurocognitive outcomes have arisen, with studies reporting delayed motor development, cognitive deficits, and behavioral alterations [6,7].

The interplay between maternal ART, immune activation, and fetal development extends beyond individual health outcomes, raising important public health questions. In high-burden regions, the cumulative effects of ART exposure, nutritional deficiencies, and limited healthcare resources could amplify the long-term risks for HEU children, impacting their ability to thrive. Moreover, the lack of standardized guidelines for monitoring and managing the health of HEU children highlights an urgent gap in global health policy [8].

**Figure 1:**
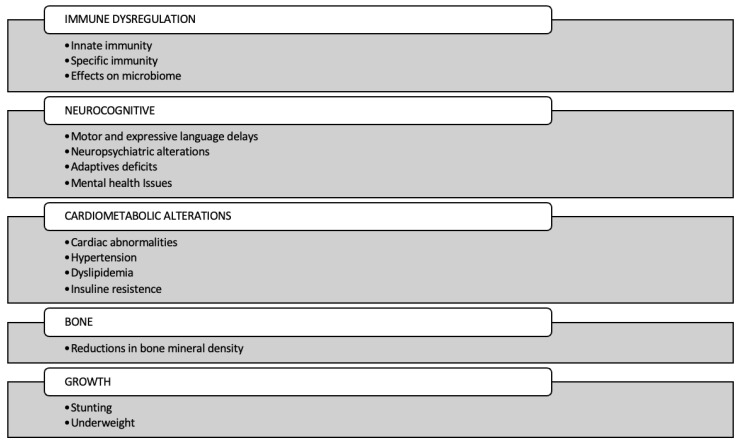
Multisystem effects of ART and HIV exposure in HEU children.

This narrative review aims to explore the multisystem effects of ART/HIV exposure in HEU children, with a particular focus on immune function, neurocognitive development, cardiovascular outcomes, growth, and bone metabolism (Figure 1).

## 2. Immune Dysregulation

### 2.1. Innate Immunity

Over the past few years, significant innate immunity alterations have been described in HEU children. Neutrophil counts remain low until age 8, with defective oxidative burst activity [9,10]. Monocyte activation is elevated at birth but normalizes by age 1, while increased oxidized LDL levels in the first 6 months contribute to oxidative stress [11]. At 6 weeks, C-reactive protein levels in HEU infants are comparable to those in HIV-infected infants and remain high until 6 months [12].

Natural killer cells decline and lose cytotoxicity in the first year, and dendritic cells exhibit exaggerated inflammatory responses, increasing bacterial infection risk [13]. HEU children also have fewer NKG2A+ CD56bright and CD56dim cells, which are linked to recurrent respiratory infections [14]. Additionally, they exhibit higher platelet counts, which may drive chronic inflammation and contribute to neurocognitive dysfunction via platelet-activating factor [9,15]. Yin et al. showed HEU children had elevated germinal centers, macrophages, IFN-γ-inducible chemokines, ferritin, and anti-inflammatory cytokines (IL-10, IL-1A). By 6 months, many biomarkers normalized, though some germinal centers and inflammatory markers remained altered [16,17].

### 2.2. Adaptive Immunity

HEU children exhibit significant immune system dysregulation, increasing their vulnerability. At birth, they have fewer naïve CD4+ and CD8+ T lymphocytes and more memory T cells, suggesting substantial antigen exposure in utero. Their reduced CD4/CD8 ratio resembles that of HIV-infected children and persists into preadolescence. T helper (Th) cell differentiation is also impaired, with lower levels of Th1, Th2, and Th17 subsets and an increase in undifferentiated Th0 cells [18].

B cell function is similarly affected, with increased apoptosis and fewer tissue memory cells during early life [19,20]. Between 6 and 14 weeks of age, Afran et al. observed reduced proportions of tissue memory and immature-transitional B cells, indicating disrupted B cell homeostasis in HEU infants [21].

### 2.3. Effects on the Microbiome

The gut microbiome in HEU children differs significantly from that of HUU infants, with more pro-inflammatory species like *Prevotella* and *Pseudomonas* [2]. HEU children also show higher levels of *Blautia*, *Shigella*, and *Klebsiella*, which are linked to inflammation, while HUU neonates have more beneficial bacteria like *Bifidobacterium breve* and *Bacteroides thetaiotaomicron* [3]. Variations in human milk oligosaccharides (HMOs) from mothers living with HIV further impact microbial colonization, worsening immune dysregulation [22].

Breastfeeding is a key factor influencing microbiome composition, primarily due to the presence of human milk oligosaccharides (HMOs), which selectively promote the growth of beneficial bacteria. However, emerging evidence suggests that the gut dysbiosis observed in HEU children is not solely attributable to differences in feeding practices. Studies by Byrne et al. and Machiavelli et al. indicate that microbiome alterations in HEU infants are also driven by in utero exposure to HIV, maternal immune dysregulation, and antiretroviral therapy. Additionally, HIV-infected mothers have been shown to produce HMOs with a distinct composition compared to HIV-negative mothers, potentially exacerbating microbiome dysbiosis in HEU children regardless of breastfeeding status.

These findings highlight the multifactorial nature of gut microbiome alterations in HEU infants, suggesting that factors beyond breastfeeding—including maternal immune activation and exposure to antiretroviral drugs—may play a crucial role in shaping microbial composition.

### 2.4. Inflammation

The complex interplay between immune dysregulation and inflammation in HEU children highlights the multifaceted nature of their increased susceptibility to morbidities. Monocytes display heightened activity, producing elevated levels of pro-inflammatory cytokines, such as TNF-α, IL-6, and IL-12 [23]. Enhanced CCR2 expression in monocytes within 4 days of birth in HEU children may facilitate their migration across the blood-brain barrier, potentially affecting neurological outcomes [24]. Similarly, the elevated oxidative stress markers, such as oxidized LDL, and altered cytokine profiles provide a pro-inflammatory milieu [11].

There are well-established differences in lymphocyte activity and development between HEU and HUU children. Maternal HIV infection significantly influences T cell subsets in the decidua, villous tissue, and umbilical cord blood, with the placenta potentially serving as a conduit for these maternal immune alterations [25]. Thymic hypoplasia, observed in HEU infants, likely restricts T cell production [26]. Impaired B cell maturation and memory in HEU infants weaken humoral immunity, increasing infection susceptibility and reducing vaccine efficacy [21]. These deficits are linked to elevated CD8+ Tregs, which impair T-cell responses, and reduced maternal antibody transfer. Maternal-fetal inflammatory cytokines and immune activation likely contribute to these effects [10,27].

Immune system dysregulation persists into childhood, further increasing HEU infants’ morbidity. Dauby et al. highlighted the heightened risk of infectious diseases, such as group B beta-hemolytic streptococcal (GBS) disease, which manifests more severely in HEU children (e.g., with meningitis). This increased risk may stem from both a lower transfer of serotype-specific maternal GBS antibodies and innate immune deficiencies in HEU neonates [28].

Chronic inflammation and oxidative stress, resulting from persistent immune activation, may contribute not only to an increased risk of infections but also to long-term cardiometabolic and neurocognitive complications. Inflammatory cytokines and oxidative stress markers, such as oxidized LDL, have been associated with endothelial dysfunction, which promotes atherosclerosis and raises the risk of hypertension and metabolic disorders later in life. Elevated inflammatory markers, including C-reactive protein and various cytokines, have been linked to endothelial dysfunction and altered lipid metabolism, both of which are associated with cardiovascular disease [15].

Similarly, persistent immune activation and systemic inflammation can affect brain development and function. Pro-inflammatory cytokines can cross the blood-brain barrier, disrupting neuronal signaling, impairing synaptic plasticity, and increasing the risk of cognitive and behavioral deficits. These findings emphasize the need for comprehensive monitoring and targeted interventions to mitigate the long-term health consequences of in utero HIV and ART exposure [9,15].

**Figure 2:**
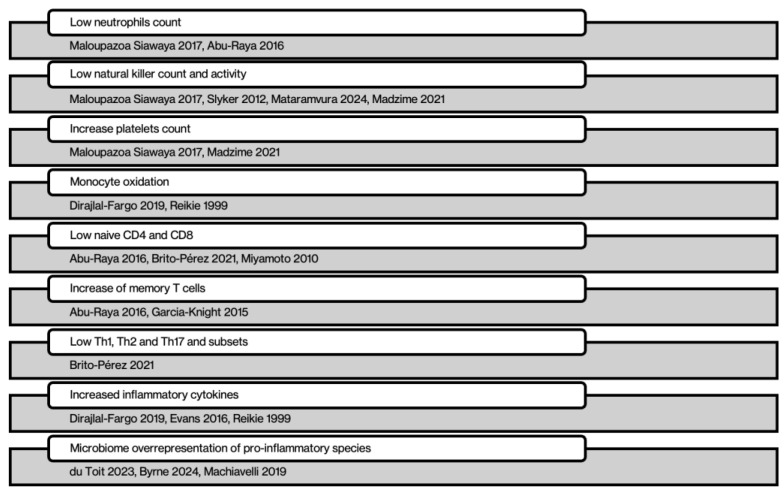
References to immune dysregulation and inflammation in HEU children [9,10,11,12,13,14,15,18,19,22,23,27].

Therefore, the extensive immunological alterations observed in HEU children highlight their increased susceptibility to morbidity and underscore the importance of understanding the interplay between innate and adaptive immunity, as well as microbiome composition, in shaping their health outcomes. These findings emphasize the need for comprehensive monitoring and targeted interventions to mitigate the long-term health consequences of in utero HIV and ART exposure. All the references about immune dysregulation and inflammation are shown in Figure 2

## 3. Cardiometabolic Alterations

Emerging evidence suggests that in utero exposure to HIV and ART may increase the risk of long-term cardiometabolic diseases, including cardiac abnormalities, hypertension, diabetes, obesity, dyslipidemia, and insulin resistance [29,30,31].

### 3.1. Cardiac Abnormalities

Chronic ART use during pregnancy might alter the intrauterine environment, predisposing the fetus to cardiac abnormalities, such as changes in left ventricular mass, septal wall thickness, and systolic or diastolic dysfunction [27,32,33]. Postnatal cardiac studies performed in HEU children showed distinct patterns of cardiac remodeling. While some studies revealed reduced left ventricular (LV) mass and increased contractility in HEU newborns up to age 2 years and preadolescents (8–12 years) exposed to ART [34,35], other studies conducted among children aged 2–7 years indicated no discernible alterations in echocardiographic measures [36]. Studies conducted on populations of children from birth to five years of age have emphasized the role of zidovudine exposure in driving specific cardiac alterations in infants, such as increased posterior wall thickness and a higher left ventricular shortening fraction [37,38,39].

A U.S.-based prospective study enrolled children ≤2 years old and conducted serial echocardiographic evaluations up to 48 months of age, comparing them to a control group of HIV-uninfected, -unexposed children (HUU). The results showed that HEU children uniformly exhibited differences in echocardiographic left ventricular diastolic indices at all ages compared to controls. These findings pointed to impaired compliance and relaxation, with a prolonged duration of isovolumic phases relative to ejection phases within the cardiac cycle, ultimately resulting in reduced cardiac efficiency. These results suggest that routine echocardiographic monitoring may be recommended for HEU children perinatally exposed to ART to ensure early detection and management of potential cardiac dysfunctions [33]. The study carried out by García-Otero et al. provides additional support for these findings. It aimed to assess the cardiovascular profile of infants exposed in utero to maternal HIV and ART compared to nonexposed infants from fetal life up to 6 months postnatally, describing cardiac concentric hypertrophy in association with maternal ZVD treatment [40].

### 3.2. Hypertension

García-Otero L et al. additionally demonstrated that HEU children exhibit significantly elevated systolic and diastolic blood pressure, with 58.8% of them meeting the criteria for elevated blood pressure compared to 7.5% of controls. Moreover, HEU displayed a thicker carotid intima-media thickness (cIMT), with half fulfilling the criteria for hypertension. Key findings revealed a significant association between maternal HIV status and infant hypertension, with maternal zidovudine treatment during pregnancy and black ethnicity also emerging as predictors [40].

The role of HIV and ART exposure in the development of hypertension was also explored by Jao J et al. through a large, prospective cohort study ongoing since 2007, focusing on HEU children from 6 to 18 years old and aiming to evaluate the safety of antenatal ART exposure on childhood and long-term outcomes. The study spans 22 clinical sites across the U.S. Analysis of blood pressure outcomes revealed that HEU children exhibited significantly higher median systolic and diastolic blood pressure Z-scores and higher rates of systolic and diastolic hypertension compared to participants in the control cohort. These differences persisted even after adjusting for potential confounders, including age, body mass index Z-scores, sex, and non-Hispanic Black ethnicity [41].

In contrast, a study conducted in Zambia between 2018 and 2019 found no significant differences in blood pressure levels between HEU and HUU children [29]. The same result emerged in a study conducted on 260 HEU 5–8-year-old children in South Africa, which analyzed blood pressure and identified that 12% of the participants had measurements above the 90th percentile. However, there were no differences in continuous measures of diastolic or systolic blood pressure, based on HIV exposure status [30].

### 3.3. Dyslipidemia

A comparison of total cholesterol (TC) and high-density lipoprotein (HDL) levels between HEU and HUU children aged 6 to 18 years who were exposed to ART in utero revealed that, although no differences were observed in HDL levels, the median TC was significantly lower in HEU group. However, no differences were found in the prevalence of high TC or in any of the other lipid subfractions. When comparing triglycerides and low-density lipoprotein, no differences were observed [41]. On the other hand, a Brazilian study that involved a small number of patients ages 6 to 11 years and sought to assess the nutritional status and metabolic changes in HEU in comparison to HUU children shows that while the nutritional status of the two groups was similar, the HEU children had higher prevalences of dyslipidemia, abnormal TC levels, elevated LDL-C, and borderline levels of both LDL-C and TC [42].

### 3.4. Insulin Resistance

A prospective cohort study conducted in Cameroon [43] provided further insight into how in utero exposure to HIV and ARV might influence infant metabolic outcomes. The study included HEU exposed to postnatal zidovudine, HEU exposed to postnatal nevirapine, and HUU. Both infants exposed to postnatal zidovudine and nevirapine were found to have lower pre-prandial insulin levels at six weeks of age compared to HUU infants. However, when plasma metabolite levels were elevated, zidovudine-exposed infants showed higher insulin levels than both nevirapine-exposed and HUU infants. These results provide valuable insights into the metabolic adaptations of HEU infants. Despite their lower pre-prandial insulin levels, the metabolite profiles and substrate utilization observed in HEU infants might reflect an altered metabolic programming influenced by in utero HIV and ARV exposure. While these adaptations may initially serve to optimize energy use, they might also predispose HEU infants to long-term metabolic complications, including insulin resistance [43].

Jao J et al. demonstrated that although insulin resistance is elevated in both HEU and HUU children from 6 to 18 years old, it is more pronounced in the HUU group [41]. This result is supported by Bengston, who found no differences in impaired glucose metabolism between HEU and HUU children from 5 to 8 years old [30].

In conclusion, significant cardiometabolic risks, including insulin resistance, dyslipidemia, hypertension, and cardiac abnormalities, are linked to in utero exposure to HIV and ART, according to the literature. These findings emphasize the critical need for ongoing surveillance, early diagnostic strategies, and targeted interventions to mitigate long-term cardiometabolic complications in HEU children.

## 4. Neurocognitive

Recent studies demonstrated a relationship among cumulative maternal HIV viremia during pregnancy, shorter durations of maternal ART (e.g., initiation during pregnancy rather than pre-pregnancy), and an increased risk of motor and expressive language delays [44,45,46,47]. Several studies have evidenced that HEU children face subtle impairments in expressive language and gross motor [7], deficits in the domain of written and spoken language [45,48,49], motor development delays [50], and deficits in mental development [6,49]. Some studies have also highlighted a higher prevalence of neuropsychiatric alterations, including internalizing problems [51] or adaptive deficits [52], with an increased risk of mental health issues. Academic performance has also shown poorer outcomes in HEU children [49,53]. However, a study conducted in South Africa assessed resilience based on longitudinal observations, finding no differences between HUU and HEU children [54].

Studies based on the correlation between neuroimaging techniques and neurologic disorders (e.g., microcephaly, seizure disorders) or cognitive dysfunction reported mixed results. Various studies identified imaging findings consistent with neurodevelopmental alterations in multiple brain sites, such as basal ganglia, hippocampal region, and cortex [52,55,56,57]. Small reductions in neuronal integrity biomarkers in HEU children appeared to partially restore over time [56], as other observations focused on improving cognitive performances [58].

A significant question is whether ART correlates with poorer neurocognitive development in HEU children. A recent meta-analysis found no associations between any specific antiretroviral and neurocognitive deficits [7]. However, some studies concluded that exposure to drugs such as efavirenz and atazanavir during pregnancy could be associated with a higher risk of neurological abnormalities in HEU patients [55,59], whereas exposure to dolutegravir appeared to produce better outcomes [60,61]. Moreover, a murine model suggested that neurological outcomes may also be influenced by the type of nucleoside reverse transcriptase inhibitor backbone of the regimen and not just by the base drug [62]. The inconsistency in findings is likely due to the diversity of assessment tools used, the wide age range of the subjects studied (from newborns to adolescents), the variety of drugs in use, and the complexity of neurodevelopment, which is characterized by rapid neurocognitive changes and influenced by multiple factors. Moreover, most studies were conducted in low-income settings, limiting their generalizability to other populations.

Most studies recognized that a key explanation for the poorer cognitive development in HEU children is the low socioeconomic status of their families [51], the presence of depression symptoms in caregivers [63], and the disparity in household wealth and maternal education [50,64]. These conditions are similar between HEU and children living with HIV [53]. Additionally, a higher risk of congenital cytomegalovirus in HEU children has been linked to the evidence of neurocognitive deficits [65].

The interplay of biological and environmental factors suggests that these neurodevelopmental risks are not deterministic. Given the abundant socioeconomic confounders, more studies are required to evaluate the role of ART exposure in these outcomes. Early detection, improved medical and social support, and targeted interventions appear to significantly improve HEU developmental trajectories, emphasizing the unmet need for more inclusive healthcare policies and support programs for this vulnerable population. While ART has been instrumental in preventing HIV transmission, its potential effects on neurodevelopment remain complex and regimen-dependent. Addressing these vulnerabilities requires integrated approaches, including optimizing maternal care, early developmental interventions, and mitigating socioeconomic disparities to improve long-term outcomes for HEU children.

## 5. Bone

The effects of long-term exposure to ART on growth and bone metabolism in HEU remain poorly understood and focus mostly on the role of tenofovir disoproxil fumarate (TDF)-containing regimens. Although these regimens are generally well-tolerated in clinical settings, significant reductions in bone mineral density (BMD) have been observed shortly after initiating TDF-containing therapies, highlighting potential concerns [4]. Tenofovir, released from the prodrug TDF, is hypothesized to cause proximal tubular cell injury through inhibition of mitochondrial DNA polymerase gamma, leading to mitochondrial dysfunction, apoptosis, and impaired renal function, as evidenced in murine models and human kidney histology. This mitochondrial toxicity disrupts vitamin D metabolism and elevates parathyroid hormone levels, contributing to bone resorption, reduced bone mineral density, and associated bone toxicity [5].

A systematic review and cohort study investigating the use of TDF during pregnancy reported it to be generally safe. Nonetheless, it emphasizes the limited availability of robust data and underscores the need for additional prospective research focusing on neonatal and infant renal and bone health [66]. Despite these concerns, TDF-containing regimens are widely accepted as first-line treatment for pregnant women living with HIV, as endorsed by the European AIDS Clinical Society (EACS) guidelines [EACS Guidelines]. Maternal TDF use from 28 weeks gestation to 2 months postpartum to prevent HBV transmission showed no significant effect on BMD in mother-infant pairs a year after delivery [67]. Similarly, other studies carried out in Malawi and Brazil found no differences in bone turnover markers or radiographic evidence of bony abnormalities associated with short peripartum TDF exposure in infants [68,69]. However, a pilot study indicated a trend toward lower whole-body bone mineral content (BMC) at 6 months of age in TDF-exposed infants compared to unexposed infants [70], with a 12% reduction in BMC in neonates exposed to TDF during the third trimester of pregnancy compared to those on non-TDF regimens, raising concerns about potential bone effects [71]. Similarly, lumbar spine bone mineral content by week 26 was lower in infants exposed to maternal TDF compared to maternal nevirapine (NVP), with a difference of -0.13 g, although the authors highlighted that the result is unlikely to be clinically meaningful [72]. The recently published Surveillance Monitoring for Antiretroviral Therapy and Toxicities (SMARTT) study found that third trimester in utero exposure to TDF in HEU neonates was associated with more rapid declines in phosphate reabsorption and lower serum 25(OH)D levels compared to unexposed neonates. No significant differences were observed for other biomarkers, highlighting the potential impact of TDF on phosphate and vitamin D metabolism [73].

When evaluating breastfeeding among mothers living with HIV, evidence from a large study conducted in Malawi suggests that prolonged exposure to efavirenz (EFV) and TDF through breast milk does not result in significant growth impairments or adverse skeletal outcomes in infants and no bone fractures were observed during the study period [74].

Long-term exposure to ART, particularly TDF-containing regimens, raises concerns about potential impacts on growth and bone health in HEU children. However, data on the long-term effects remain limited, with most findings indicating minimal clinical significance for growth and skeletal outcomes. Longitudinal research remains essential to assess the potential long-term risks of osteoporosis and fractures in HEU children [75].

## 6. Growth

Emerging evidence suggests HEU children may face a heightened risk of growth and developmental impairments. Several factors have been proposed to explain these vulnerabilities, including immunologic abnormalities arising from repeated microbial exposure, potential adverse effects of antiretroviral medication exposure, and socioeconomic challenges [76].

### 6.1. Linear and Ponderal Growth

Linear growth is an important reflection of overall child well-being. Disparities in linear growth have been observed between HEU and HUU children [76,77,78]. A longitudinal study conducted in Zimbabwe sought to evaluate growth differences between HEU and HUU infants. The cohort included infants enrolled at six weeks of age and followed for six months, during which exclusive breastfeeding was maintained. The study reported significantly lower mean weight-for-age (WAZ) and length-for-age (LAZ) z-scores among HEU infants compared to HUU infants from birth to 16 weeks. Conversely, mean weight-for-length z-scores (WFLZ) were notably higher in HEU infants than in HUU infants. While body mass index-for-age (BMIAZ) z-scores showed no significant differences between the two groups at birth and at 16 weeks postpartum, a significant disparity emerged at six weeks of age, with HEU infants exhibiting higher mean BMIAZ z-scores compared to HUU infants [79].

The findings are corroborated by a Nigerian study, which examined growth trajectories in HEU and HUU infants from birth to 18 months of age. The study reported that HEU infants exhibited significantly lower WAZ, LAZ, and head circumference-for-age (HCAZ) z-scores at birth compared to their HUU counterparts, but there were no statistically significant differences for BMIAZ and WFLZ [76]. Other studies carried out in Zambia [80], Malawi, and Uganda [77,81] also reported impairments in ponderal and linear growth indices among HEU children during the first 2 years of life.

### 6.2. Stunting and Underweight

Stunting and underweight refer to impaired growth and development in children resulting from inadequate nutrition, frequent infections, and insufficient psychosocial stimulation. A child is classified as stunted when their LAZ is more than two standard deviations below the WHO Child Growth Standards median and is defined as underweight when WAZ falls more than two standard deviations below the median of the WHO Child Growth Standards [World Health Organization]. A study performed in Kenya on a large population of children evaluated at 6 weeks and 9 months revealed a significant association between HIV exposure and a higher prevalence of stunting. At 6 weeks, 34% of HEU children were stunted compared to 18% of HUU children. Similarly, at 9 months, 20% of HEU children were stunted compared to 10% of HUU children. Regarding weight, HIV exposure was not found to be associated with differences in mean WAZ at 9 months or with the prevalence of underweight at 6 weeks or 9 months [82]. Analogous findings were reported in a study by Aizire, which investigated populations of children with prolonged perinatal exposure to maternal HIV and ART in Uganda and Malawi. The study evaluated children at 12 and 24 months of age and found that stunting at 24 months was prevalent among HEU children, affecting 50% in Malawi and 30% in Uganda [77]. Two recent prospective cohort studies conducted in Nigeria and South Africa reported similar growth faltering trends among HEU children compared to HUU children through 18 and 12 months of age [76,83]. Another study shows how, in rural Zimbabwe, where stunting prevalence was notably high, HEU children demonstrated almost double the rate of stunting compared to HUU children. Comparable disparities were observed in underweight prevalence [64].

A significant question is the extent to which HIV infection and exposure to ART contribute to impaired growth and the increased risk of stunting and underweight. First, a substantial immunological deficit was noted in HEU, which was mostly ascribed to long-term immune activation brought on by exposure to HIV [10]. They might be more susceptible to congenital and postnatal infections, which could have a negative effect on their growth [84].

ART exposure during pregnancy and breastfeeding can be considered as another potential factor. A Malawian study found that 88% of HEU children had antenatal ART exposure and 91% via breast milk, despite the general recommendation against breastfeeding for HIV-positive mothers [85]. While studies link HIV and ART exposure to stunting risk [78,83,86], maternal ART timing and viral load seem not to be significant predictors of stunting at 24 months. Instead, factors like inadequate feeding practices and food insecurity were key contributors [87].

In Malawi, 20% of HEU mothers were underweight compared to 7% of HIV-negative women, likely affecting breast milk quality [85,88]. Supporting ART’s direct impact, a Botswana study found lower LAZ and WAZ scores in HEU children exposed to combination ART versus zidovudine monotherapy through 24 months [89].

Growth deficiencies in HEU children are not purely deterministic, as suggested by the interaction of biological and environmental factors. Given the multiple socioeconomic and nutritional confounders, further research is necessary to isolate the direct impact of ART exposure on growth outcomes. Early nutritional interventions, healthcare access, and improved social support appear to play a crucial role in mitigating these risks.

While ART helps prevent HIV transmission, addressing growth deficits requires a holistic approach that integrates medical, nutritional, and socioeconomic strategies to improve long-term outcomes for HEU children.

## 7. Conclusions

HEU children often receive limited medical, nutritional, and social support compared to children living with HIV, who generally benefit from comprehensive care [6], and no specific guidelines are available for durations or specific follow-up.

The critical role of ART in preventing mother-to-child transmission of HIV cannot be overstated. ART has been a transformative tool in reducing vertical transmission rates globally, significantly lowering the number of new pediatric HIV infections. It has enabled millions of children to grow up HIV-negative, a success that has fundamentally changed the landscape of HIV prevention worldwide. However, while ART has successfully reduced the incidence of HIV infection in HEU children, their health outcomes remain a concern, requiring a focus on comprehensive care for these children.

Interventions specifically directed toward this population have shown a sensible improvement in neurodevelopment outcomes [90], and targeted strategies in other areas—such as cardiovascular and bone health monitoring, growth, and immunity control—could be beneficial in addressing their health challenges. In resource-limited settings, the implementation of structured screening and intervention programs is critical. Regular neurodevelopmental assessments, nutritional supplementation programs, and early childhood stimulation initiatives have demonstrated positive outcomes in similar populations. Additionally, community-based healthcare models integrating maternal support, mental health services, and socioeconomic assistance could play a pivotal role in improving HEU children’s overall well-being. Furthermore, studies highlight significant cardiometabolic risks associated with in utero exposure to HIV and ART. Integrating routine cardiovascular assessments, lipid and glucose metabolism monitoring, and early lifestyle interventions into HEU healthcare frameworks could help prevent future metabolic disorders. 

In conclusion, while ART remains indispensable in reducing vertical HIV transmission, developing international guidelines for HEU follow-up care, with clear recommendations on periodic evaluations and targeted interventions, remains an urgent need to bridge existing healthcare gaps and ensure a more equitable standard of care for this vulnerable population.

## 8. Search Strategy, Selection Criteria

A search was conducted using MEDLINE and Google Scholar to identify peer-reviewed, English-language studies published up to 30 November 2024, examining inflammation, neurocognitive development, cardiovascular health, bone health, and growth outcomes in HEU individuals. Relevant references were identified through a combination of search terms, including: “HIV exposed uninfected” AND “inflammation” OR “neurodevelopment” OR “cognitive” OR “neurological” OR “cardiac” OR “cardiovascular” OR “metabolic” OR “lipid” OR “hypertension” OR “bone” OR “growth.” Studies were selected through a two-step process comprising an initial screening of titles and abstracts followed by a detailed full-text review of the shortlisted articles. This selection process was independently undertaken by five authors, with any disagreements resolved through discussion and consensus. It is important to note that this work is not a systematic review. Instead, the final selection of references was curated based on multiple criteria, including publication date, originality, accessibility, and relevance to the scope of this narrative review. To enhance the rigor and reliability of the review, the corresponding author applied the Scale for the Assessment of Narrative Review Articles (SANRA), a validated six-item instrument [91].
